# Food preservation by cold plasma from dielectric barrier discharges in agri-food industries

**DOI:** 10.3389/fnut.2022.1015980

**Published:** 2022-11-16

**Authors:** Hao Jiang, Qian Lin, Wenqing Shi, Xiuzhu Yu, Shaojin Wang

**Affiliations:** ^1^College of Food Science and Engineering, Northwest A&F University, Xianyang, China; ^2^Shanxi Rural Science and Technology Development Centre, Xi’an, China; ^3^College of Mechanical and Electronic Engineering, Northwest A&F University, Xianyang, China

**Keywords:** cold plasma, dielectric barrier discharges, agri-food, food components, decontamination

## Abstract

**Background:**

Cold plasma (CP) can be defined as partially or wholly ionized gas carrying myriads of highly reactive products, such as electrons, negative ions, positive ions, free radicals, excited or non-excited atoms, and photons at ambient temperature. It is generated at 30–60°C under atmospheric or reduced pressure (vacuum). In contrast to thermal plasma, it requires less power, exhibits electron temperatures much higher than the corresponding gas (macroscopic temperature), and does not present a local thermodynamic equilibrium. Dielectric barrier discharges (DBD) are one of the most convenient and efficient methods to produce CP.

**Scope and approach:**

Cold plasma technology has the potential to replace traditional agri-food processing purification methods because of its low energy requirements and flexible system design. CP technology works by reducing bacteria levels and removing pests and mycotoxins from your produce at harvest. It can also catalyze physiological and biochemical reactions and modify materials. It can meet microbial food safety standards, improve the physical, nutritional, and sensory characteristics of the products, preserve unstable bioactive compounds, and modulate enzyme activities. This manuscript also discusses the quality characteristics of food components before/after CP treatment.

**Key findings and conclusion:**

In the past decade, CP treatments of food products have experienced increased popularity due to their potential contributions to non-thermal food processing. There is no doubt that CP treatment is a flexible approach with demonstrated efficacy for controlling many risks across food and agricultural sustainability sectors. In addition, CP technologies also can be applied in food-related areas, including modification of chemical structures and desensitization treatments. There is a need to fully assess the benefits and risks of stand-alone CP unit processes or their integration as a processing chain as soon as the economic, ecological, and consumer benefits and acceptability are considered.

## Introduction

Microorganisms, often known as microbes, are minute organisms that can be single cells or colonies of cells. Food corruption and human disease can be contributed to microorganism contamination in food as one of the major reasons. Unwanted microorganisms because of food spoilage and food pathogens can cause illness, especially if the food is prepared or kept incorrectly. Microbial toxins and spores are also possible contaminants of food. However, in the process of preparing food, microbes are diminished or prevented by preservation or processing techniques including cooking, utensil hygiene, or low temperatures. An autoclave to control germs with heat and pressure is utilized when total sterility is required, such as with surgical equipment. Of these, the usage of radio frequency (RF) heat treatment is proposed for inactivating microorganisms not just because it is an effective method of preventing food spoilage, but also because it can create multifarious delicacies under certain conditions. Thermal processing technology has been widely used in agriculture, food and industrial production fields, its functions include sterilization, preservation, deworming, drying, cooking and modification (1–7). However, considering the amount of energy and the quality of heated food, heating is not always suitable due to sensitivity to food quality. At the same time, overcooking, lowering nutritional value, and changes in flavor and sensory attributes caused by overheating of foods may also destroy the acceptability of consumers. The modern food industry is also searching for methods to meet the growing trend for healthy and nutritional food with “fresh” attributes (8). On the other hand, in many places, regulatory reviews of agricultural and food production inputs are being conducted in order to ensure their long-term sustainability, human safety, and environmental safety. Many safety issues include persistent contamination, spoilage, parasite, agricultural chemical pollution, and antibiotic contamination in the agricultural and food sectors. Therefore, new strategies for hazard control in the food and agricultural industries as well as in healthcare are needed. Additionally, food manufacturers are looking for strategies to lessen or reduce allergy, either in foods or in processing settings, since the safety of foods in terms of their immunological reactivity is becoming increasingly relevant.

After solids, liquids, and gases, plasma is referred to as the fourth state of matter in science. It is a gaseous substance that has been electrically electrified and is made up of charged particles, free radicals, and some radiation. A partially or completely ionized gas, made up of ions, free electrons, atoms, and, most significantly, photons in their fundamental or excited states, is produced during an electrical discharge to produce plasma. These are classified as either “heavy” or “light” species (photons and electrons) (9). Usually, we can observe a homogeneous glow or filamented structure. According to the mechanism of creation, the plasma may be divided into two classes: equilibrium (thermal plasma) and non-equilibrium (low-temperature plasma) (10). Thermal plasma consists of ions, electrons, and gas molecules in thermodynamic equilibrium created by thermonuclear fusion at temperatures of about 20,000 K. Such a kind of temperature can hardly be utilized by industries or home use. The low-temperature plasma is usually less than 150°C, between which quasi-equilibrium plasma (100–150°C) and non-equilibrium plasma (<60°C) can be distinguished. Local thermodynamic equilibrium between species, such as electrons and gas molecules, is present in the quasi-equilibrium plasma. Without any local thermodynamic equilibrium, partial ionization in the non-equilibrium plasma results in lower temperatures for the gas molecules and higher temperatures for the electrons, which lowers the system’s overall temperature. The definition of plasma as an ionized (partially or completely ionized). Cold plasma (CP), in contrast to thermal plasma, is produced at atmospheric pressure or lower pressures, using less power. It also shows electron temperatures that are significantly greater than the comparable gas and lacks a local thermodynamic equilibrium (11).

Because the ions and uncharged molecules only receive a little amount of energy and maintain a low temperature, CP is appropriate for treating food products that are sensitive to heat. It can satisfy microbiological food safety requirements, enhance the goods’ physical, nutritional, and sensory qualities, protect unstable bioactive chemicals, and control enzyme activity. Food is a complex system, which needs to argue the main components’ alternation before/after CP treatment. However, there is little systematic information to summarize the impact of CP treatment on changes in food components. As a result, the latest advancements in CP for food storage are summarized in this article and explore the influence on quality characteristics of food components during/after CP treatments. The opportunities and challenges of CP applications are also discussed.

## Fundamentals and mechanisms

### The purpose of the cold plasma in food applications

Cold plasma technology is a fast and non-invasive treatment, which has the potential to replace or work in conjunction with several production phases in the agricultural and food sectors, including lower bacterial levels at harvest (12), pesticide degradation (13, 14), pest and mycotoxin elimination (15), non-thermal pasteurization/sterilization of food (16), decontaminant (17), and catalysis/modification (18, 19), due to the flexible system architecture and low energy needs. Furthermore, due to its acidic environment, which alters the redox potential and conductivity and leads to the generation of reactive oxygen and nitrogen species, plasma-activated water can be used as an alternate technique for microbial inactivation. It is discovered that *Pseudomonas fluorescens* was responsive to plasma-activated water treatment, and after 3 min of CP treatment, it was decreased to below detection limits (20). For instance, Patange et al. (17) used plasma-activated water to control *Listeria innocua* and *P. fluorescens* inoculated on lettuce and found that *P. fluorescens* was responsive to plasma-activated water treatment and after 3 min of CP treatment, it was decreased to below detection limits. 2.4 Log10 CFU/g less *L. innocua* were present after 5 min of CP treatment. Liao et al. (21) used ice made from plasma-activated water for shrimp preservation. The results indicated that the deteriorating changes in color characteristics and hardness were delayed and the volatile basic nitrogen (TVBN) was reduced to below 20 mg/100 g. The pH of shrimps treated with plasma-activated water or ice remained below 7.7 during storage, which made the extending storage time by 4–8 days. CP was also used to reinforce the physical-chemical properties of edible film. Chen et al. (22) studied the functional properties of zein film enhanced by chitosan and CP treatment. After CP treatment, oxidation and etching cause the protein molecules to unfold, exposing the internal molecular groups and enhancing the crosslinking of the proteins, resulting in a significant enhancement of the mechanical behaviors of the film. Romani et al. (23) developed a fish protein film with low water sensitivity for food preservation. After CP treatment, a decrease in water vapor permeability and solubility can be observed obviously. CP was also used to improve antioxidant activities. The strengthening effect of freshly cut pitaya fruit has increased antioxidant activity and phenolic build-up, which was investigated by Li et al. (24). The results indicated that CP treatment had the capacity to increase the antioxidant activity and phenolic build-up in freshly cut pitaya fruit, due to its ability to alter the relative gene expression. This CP treatment could be used for changing the consumption of primary sugars, raising the energy level, enhancing the signaling function of reactive oxygen species (ROS), and triggering the metabolism of phenyl propanoid in freshly cut pitaya fruit. In addition, CP treatments are also used for enzyme inactivation (25).

### Mechanisms of microbial decontamination by cold plasma in foods

Non-thermal processes can inhibit microbial growth, degrade the contaminations, and meanwhile enhance the items’ dietary, sensory, and physical qualities, which can be used for unstable bioactive compounds preserving and enzyme activity modulating. As an emerging non-thermal technology, mechanisms of CP treatments for food sterilization are complex and not completely understood. Most of the studies available are focused on the indirect mechanisms of microbial destruction (26). Fortunately, CP has been proven to decontaminate efficiently the food with a minimal impact on product quality, as growing studies are available on the antimicrobial efficacy of CP. The summarized mechanisms of microbial decontamination by CP in foods are listed in Table 1.

**TABLE 1:** Mechanisms of microbial decontamination by cold plasma (CP) in foods.

	Reactive species	Mechanisms	References
Charged particles	Electrons, atomic, or molecular ions, etc.	1. Membrane lipid of microorganisms peroxided. 2. Amino acid in proteins of microorganisms denaturized. 3. Breaking structural bonds in cell wall component peptidoglycan. 4. Permeabilization of the cell membrane or cell wall. 5. Damaging iron–sulfur and mononuclear iron enzymes. 6. Changing the pH that microorganisms rely on.	(10) (26) (28) (27) (9, 25) (29)
Reactive oxygen species (ROS)	alkoxyl (RO⋅), carbonate anion radical (CO_3_⋅–), hydrogen peroxide (H_2_O_2_), hydroperoxyl (HO_2_⋅), hydroxyl radical (⋅OH), ozone (O_3_), peroxyl (ROO⋅), superoxide anion (O_2_⋅–), and singlet oxygen (1O_2_). Common examples of RNS in humid air plasma are included.		
Reactive nitrogen species (RNS)	alkylperoxynitrite (ROONO), nitric oxide (NO⋅), nitrogen dioxide radical (⋅NO_2_), peroxynitrite (ONOO–), and peroxynitrous acid (OONOH).		
Ultraviolet rays	Electromagnetic radiation created by aero-ionization	Directly damaging genetic materials and inhibiting the replication of DNA. Formation of thymine dimer due to photon emissions, modifications of nucleo-bases, and nucleotide oxidation by reactive species.	(30, 31)
Gene expression changing		Activating phenylpropanoid metabolism in fruits and vegetables. The metabolism can be used for microorganism inactivation.	(24)
Plasma-activated water treatment	Creating the acidic environment water full of ROS and RNS	Similar to reactive species.	(20)

The action electronic impact (excitation, vibration, dissociation, attachment, and ionization), ion-molecule reactions, ion-ion neutralization, penning ionization, quenching, neutral chemistry, and three-body neutral recombination, in addition to photoemission, photo-absorption, and photo-ionization by UV and photons, are responsible for the generation of active species in a CP (25). The oxidation of proteins and DNA by active substances such as reactive oxides and nitrides makes low-temperature plasma excellent for sterilization. The gas between the plate electrodes, the plasma source’s design, the power applied to the gas, vacuum, treatment time, and humidity levels are only a few examples of variables that might affect the reactive species and their concentrations in the plasma (27).

### Sources of cold plasma

**FIGURE 1:**
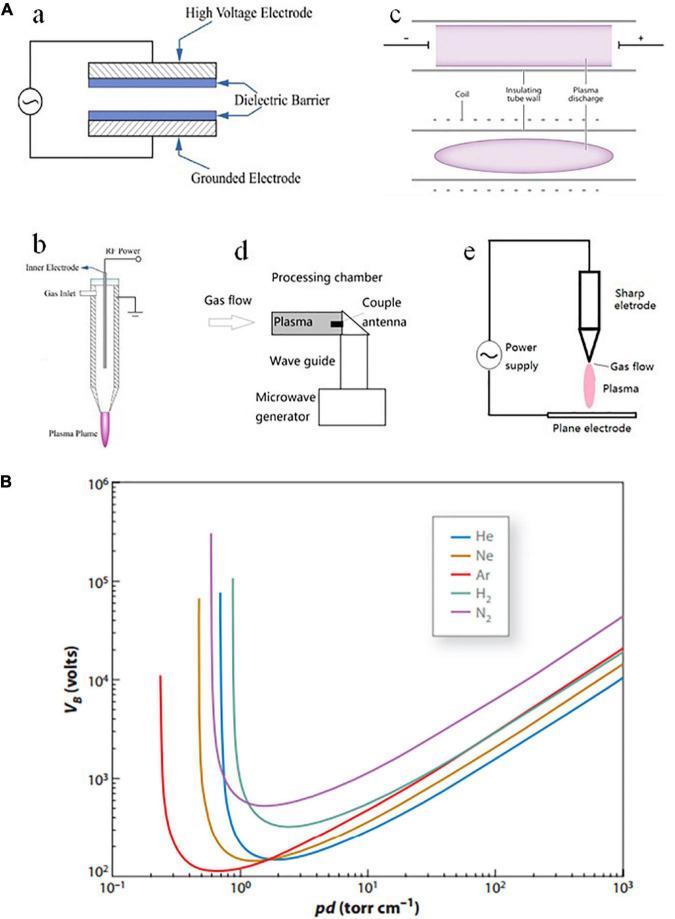
**(A)** Schematic diagram of different plasma sources: (a) dielectric barrier discharge, (b) plasma jet, (c) radio frequency discharge, (d) microwave discharge, and (e) corona discharge plasma jet (25, 35, 36). **(B)** Paschen ionization curves obtained for helium (He), neon (Ne), argon (Ar), hydrogen (H2), and nitrogen (N2). VB (breakdown voltage, in volts) as a function of pd (pressure × distance, in torr cm^–1^) under parallel plate electrodes (39).

Plasma is an ionized gas that contains a variety of active species with net neutral charges. However, it is of particular interest for use in the food sector since it may be useful for processing food at low temperatures. It is believed that non-thermal plasma was formerly created at low pressures and power levels. As technology develops, plasma generators that can function at atmospheric pressure have been developed thanks to recent advancements in plasma engineering. The plasma source mainly includes dielectric barrier discharges (DBD), gliding arc discharge, corona discharge plasma jet, and microwave/radio frequency plasma (Table 2 and Figure 1). DBD is a non-equilibrium gas discharge in which an insulating medium is inserted into the discharge space. The gliding arc discharge is caused by a high voltage applied by the power supply to the two electrodes causing an electrical breakdown of the gas flowing between the electrodes at the narrowest part of the electrodes. A strong arc of high current is generated, and the arc is extinguished and then re-activated, and the cycle repeats. A corona discharge is a local self-sustaining discharge of a gaseous medium in an inhomogeneous electric field. A plasma jet is two coaxial electrodes with the gas discharging between them at high flow rates. Microwave/RF plasma is a low-temperature plasma produced by ionizing the air around the electrodes using high frequency and high voltage.

**TABLE 2:** The classification of plasma sources.

CP	Generating mechanism and characteristics	References
Dielectric barrier discharges (DBD)	Plasma occurs between two electrodes, and then, an AC high voltage is applied on the electrodes. It is an excellent CP source with 1–10 eV and high density.	(32)
Gliding arc discharge	Two (or more) metallic electrodes connected to an AC or DC high-voltage transformer. A plasma plume is generated when the high voltage is applied. This arc is then pushed away by a gas flow and glides along the electrodes until it collapses.	(33)
Plasma jet	Two coaxial electrodes, between which gas flows at high rates. The free electrons are accelerated by the RF field and collide with molecules of background gas. These inelastic collisions can produce various reactive species (excited atoms and molecules, free radicals) that exit the nozzle at high velocity.	(25, 34)
Corona discharge plasma jet	Containing substantial electric field for expediting the ionization energy of arbitrarily produced electrons to that of milieu gas atoms or molecules.	(29)
Microwave (MW)/radio frequency (RF) plasma	RF plasma is usually achieved when a gas is placed within an oscillating electromagnetic field, produced by an induction coil or distinct electrodes kept outside the reactor. MW/RF CP is usually generated under reduced pressure.	(35, 36)

### Factors influencing the efficiency of cold plasma

#### Atmospheric and reduced pressures

Some decades ago, the application of plasma-related technologies in the food industry was rare due to the low-pressure level (>105 Pa) input, the propagation power of up to 50 MW, and the extremely high temperatures generated. However, contemporary innovations in plasma engineering make a possible application of plasma sources that can operate at atmospheric pressure and produce mild temperatures.

Both atmospheric-pressure (AP) and low-pressure (LP) plasmas have been applied to food research fields. AP plasma systems are easy to build due to no airtight vacuum chambers required. Thus, the materials can be moved through a treatment zone easily. However, LP plasma systems encounter technological challenges concerning treatment speed and throughput volume that prevent them from meeting the criteria for commercial scale-up processing (37). Although, LP plasma systems encounter technological challenges regarding treatment speed and throughput volume that prevent them from meeting the criteria for commercial scale-up processing. Contrary to LP plasma, AP plasmas are difficult to ionize and do not emit UV radiation at large dosages because air absorbs UV rays at AP (36).

The arrangement of the distance between the electrodes (the gap width) and the gas pressure between them defines the ionization voltage for any gas combination (38, 39). Figure 1B shows lower pressure of the gas also results in a reduction in the voltage needed to ionize it, which is the relationship’s impact for different gases. Using reduced pressure treatment chambers to create plasma and transfer it to the food surface to be treated, several CP generating technologies have been developed as a result of this essential property of ionization potentials. In addition to the throughput restrictions imposed by batch processing, it is essential to remember that not all food products can withstand vacuum conditions. Materials to be treated can be conveyed through a treatment zone *via* a conveyor without the requirement for vacuum-tight chamber doors or gaskets. But as shown in Figure 1B, ambient pressure processing presents further difficulties for CP systems, which makes us have no choice but to raise the voltage input or limit the distance between two electrodes.

#### Modified atmosphere

Modified atmosphere packaging (MAP) is widely used for food preservation. The features of CP could be used for MAP food sterilization. As the constitution of gas would influence the ionization, the MAP food sterilized by CP needs painstaking research. Han et al. (40) studied the performances of food preservation treated by CP under three kinds of MAP conditions, namely, 70% N_2_ + 30% CO_2_, 90% N_2_ + 10% O_2_, and 70% O_2_ + 30% CO_2_. The results indicated that ROS production, in-package inactivation effectiveness, and post-treatment storage duration were all positively impacted by the oxygen levels in the user’s working gas. After 15 s of treatment with a high-oxygen MAP mix and 24 h of post-treatment storage, *Listeria* populations were undetectable. However, the production and impact of RNS were influenced by oxygen levels in addition to nitrogen content. Strawberry CP decontamination was examined by Misra et al. (41) using two different gas mixes within a sealed packaging. The outcomes showed that the plasma treatments with the two gas mixes had comparable effects on the levels of microbial decrease. In other words, the micro-flora of the strawberries decreased from the starting values of 5 log_10_ CFU/g after 300-s treatments by an average of 3 log_10_.

#### Attributes of microorganisms

It has been shown that plasma has a tremendous antibacterial effect, inactivating both Gram-positive and Gram-negative strains of bacteria, yeasts, and even viruses. Plasma targets and destroys various structures of microorganisms, etching cell walls, disrupting biofilms, and peroxidizing lipids, and bacterial DNA and RNA may be affected by oxidative damage, base modifications, and strand breaks. In addition, large molecules (e.g., proteins) may be unfolded or modified, all of which are specific mechanisms of plasma sterilization. Currently, low-temperature plasma disinfection technology has been developed and used in a variety of fields with promising results.

The features of the target microorganisms are crucial for CP technology decontamination success, which are given in Table 3. Some studies have used DBD to inactivate the *Bacillus subtilis* spores in culture media (42). Bourke (43) figured out that mono-species surface inoculations had greater inactivation rates than seed native micro-flora, which appear as multispecies microbial communities dispersed on the surfaces and within internal seed structures. It is also reported that different modes of interaction of ROS and RNS created by CP treatment with Gram-positive and Gram-negative bacteria are observed (40).

**TABLE 3:** Microorganism inactivation with cold plasma (CP) technologies.

Microorganism	Materials	Plasma system	References
*Bacillus sp*.	Blueberry juice	Plasma jet	(44)
*Bacillus subtilis* spores	Culture media	Dielectric-barrier discharge CP	(42)
*Bacillus cereus*	Red pepper flake	Microwave discharge plasma system	(45)
*Escherichia coli* O157:H7	Tryptic soy broth	Dielectric barrier discharge CP	(46)
	Bulk Romaine lettuce	Dielectric barrier discharge CP	(47)
	Onion flakes	Moisture vaporization combined helium dielectric barrier discharge cold plasma	(48)
	Baby kale leaves	Dielectric barrier discharge CP	(49)
	Tray sealer with PBS suspension	In-package modified atmospheric CP	(40)
*Listeria monocytogenes*	Cabbage, lettuce, and dried figs	Microwave-powered CP	(16)
	Ready-to-eat ham	Dielectric barrier discharge CP	(50)
	Dry-cured beef	Argon and oxygen gas electrodischarge plasma system	(51)
	Tray sealer with PBS suspension	In-package modified atmospheric CP	(40)
Microorganisms, psychrotrophs, yeast, and mould	Pork	Low-pressure CP	(52)
*Salmonella*	Coconut water	High-voltage atmospheric CP	(53)
	Brain heat infusion broth for assay	Dielectric barrier discharge CP	(54)
	Cherry tomato	Microwave-powered CP treatment	(55)
	Bulk grape tomato	In-package modified atmospheric CP	(56)
	Thyme essential oil/silk fibroin nanofibers	Nitrogen flow CP system	(57)
*Staphylococcus aureus*	Tray sealer with PBS suspension	In-package modified atmospheric CP	(40)
Total aerobic bacterial counts	Fresh-cut pitaya	Dielectric barrier discharge CP	(24)
	Pork	Dielectric barrier discharge with MAP	(58)

## Impact of cold plasma on characteristics and chemical changes of food

Researchers are also very interested in how foods behave throughout CP therapy and how physical qualities and chemical changes are related. Making clear the relationship between the physical and chemical indexes of each food component after CP treatment can offer guiding significance on food processing and preservation.

### Cold plasma applied to water

Water-based food, such as beverages, can be easily infected with microorganisms. Fortunately, CP is effective in water sterilization. However, the interaction of water and components of beverages requires certain tests to confirm the CP effects. Hou et al. (44) studied the effect of blueberry juice treated by CP jet. After reaching the equal sterilizing thermal effect, the content of phenolics significantly increased by CP treatment, which could better preserve the original color of blueberry juice. It was chosen to have considerably shorter exposure times to CP for anthocyanin and vitamin C. In studies using antioxidants, an increase in oxygen content led to rising trends in antioxidant activity in DPPH (2,2’-diphenyl-1-picrylhydrazyl radical) and ABTS [2,2’-azino-bis-(-3 ethylbenzothiazoline-6-sulfononic acid)] assays. Chocolate milk drink treated by nitrogen plasma flow system in Coutinho et al. (59, 60) showed particles that were larger, more consistent and had a different melting profile than the pasteurized product (lower temperature, bound water with a greater enthalpy), which suggested denaturation processes and the creation of protein aggregates. The mild and severe conditions led to a reduction of the bioactive compounds, changes in fatty acid composition, less favorable health indices, and lower number of volatile compounds. The authors considered that the drinks that were subjected to CP and pasteurization showed various physical traits and microstructures. (61). However, CP is still an effective method for beverage sterilization. Besides, guava-flavored whey beverage (62), coconut water (53), and white grape juice (63) are also sterilized by CP technologies.

**FIGURE 2:**
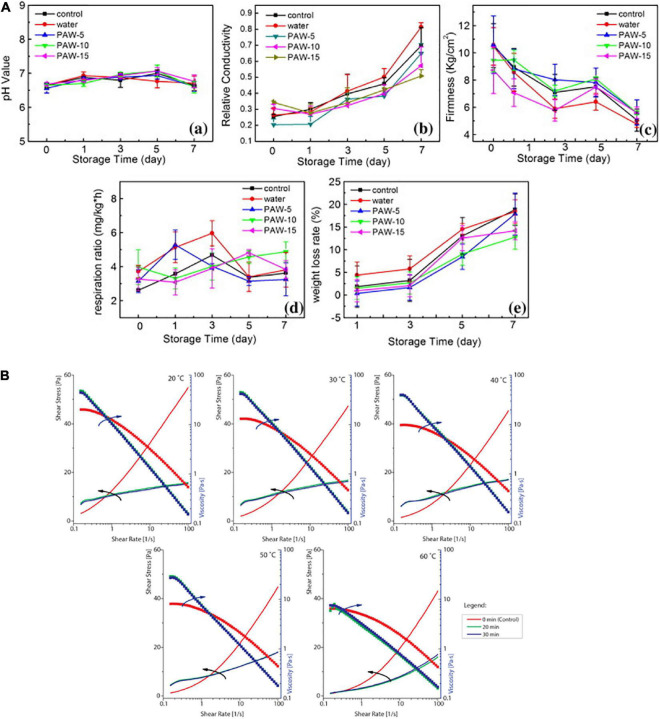
**(A)** pH values (a), relative electric conductivity (b), firmness (c), respiration ratio (d), and weight loss (e) of control, water-, PAW- 5-, PAW- 10-, and PAW-15-treated button mushroom during storage time (PAW: plasma-activated water) (64). **(B)** The viscosity profiles of 1% control and CP-treated xanthan solutions over a shear rate range of 0.1–100 s^– 1^ and over a temperature range of 20–60°C. Arrows indicate an ordinate axis corresponding to the curves (66).

Some kinds of microorganisms can also be rendered inactive by plasma-activated water. The significance of ROS in plasma-activated water solutions has been underlined by studies to date on the effects of pH and H2O2 on the development of plasma-activated water disinfection. The species produced in the fluid remain stable for a long time, which contributes to their long-lasting antibacterial qualities. For instance, Xu et al. (64) used plasma-activated water for mushroom preservation. It indicated that the plasma-activated water decreased the microbiological counts by 1.5 log for bacteria and 0.5 log for fungus during storage. Additionally, the relative electrical conductivity, observed hardness, and observed respiration rate revealed that plasma triggered water soaking and postponed mushroom softening. Meanwhile, no significant change was observed in the colour, pH, or antioxidant properties of *A. bisporus* treated with plasma activated water (Figure 2A). Lettuce (17) and shrimps (21) are also treated with plasma-activated water for preservation.

### Cold plasma applied to carbohydrates

Starch and saccharides, which include saccharose, fructopyranose, and amylaceum, among others, are the most prevalent types of carbohydrates found in plant tissues. Most plants synthesize carbohydrates to store energy. In comparison with foods such as potatoes, wheat, maize, rice, and cassava, it is also the most prevalent in human diets. In carbohydrates, starch is one of the most important compounds existing in food tissues.

Bulbul et al. (65) reported the effect of bell-jar-type CP treatment on xanthan gum at different powers and treatment times by a bell-jar-type CP system. They found that the CP treatment reduced mass, tapped density, and compressibility index while increasing the porosity ratio and angle of repose. It had no influence on the proximate composition of xanthan gum. The etching phenomenon of the CP treatment increased the surface area, according to the Brunauer–Emmett–Teller (BET) study. Moreover, the CP treatment also caused alterations in pH, hydroxyl value, and acidity. Misra et al. (66) used DBD CP to improve the viscosifying and emulsion-stabilizing properties of xanthan gum. In CP-treated xanthan gum, it showed more potential benefits for salad-dressing and instant dry soup formulations as it increases in its viscosifying ability at low shear rates (Figure 2B). Emulsion-stabilizing activity of the gum can be found with changes without affecting the color or the basic polysaccharide backbone of the xanthan. The performance of fructooligosaccharides (FOS) in orange juice after DBD CP treatment was investigated by Almeida et al. (67). The results indicated some changes in the polymerization degree of FOS. The treated samples did, however, exhibit a little difference in the color characteristics following both treatments, and the assessed methods also failed to degrade the orange juice’s primary organic acids, which is more desirable than high-temperature- and high-pressure-treated ones.

Native starch is also modified by CP. Gao et al. (68) found that except for the increased crystallinity, more fissures and holes appeared, granule aggregation occurred, and the starch digestibility was strongly enhanced. It works well for starch hydrolyzation processes such as brewing, food fermentation, and the manufacture of bioethanol. Okyere et al. (18) figured out that after RF CP modification, the resistant starch content of cereal and tuber waxy starches was increased, while the setback, final viscosities, and crystallinity decreased. The starch films treated by high-voltage atmospheric CP were carried out by Pankaj et al. (69), showing that all of the films underwent CP treatment, which increased the glass transition temperature, surface roughness, and surface oxygenation. Additionally, the findings unmistakably show that the amylose content and the starch source are crucial in determining how it interacts with CP. Moreover, hexamethyl disiloxane CP treatment may improve the barrier and hydrophobic characteristics of starch with 50% amylose-incorporated methyl groups and favor a small amount of water–film interaction caused by the starch components’ helix organizing abilities (70).

### Cold plasma applied to proteins

All living things contain a highly complicated molecule called protein. It contributes crucial features to the finished product, such as gelling properties, emulsifying ability, or water and oil holding capacity. It is a significant structural component of many foods. The majority of proteins fold into distinctive, three-dimensional shapes. There are four distinct parts of a protein’s structure that biochemists frequently discuss. A protein’s functionality, such as its increased solubility or emulsifying capacity, may change as a result of emerging technologies.

Many studies revealed the effect of CP treatment on proteins. Two common food ingredients, namely, hemoglobin and gelatine from pork, were exposed under a DBD CP reactor. CP treatment affected the functional properties in different ways. For instance, after CP treatment, the proteins’ solubility dramatically decreased, while their ability to retain oil improved significantly. Pérez-Andréset al. (71) agreed that CP can also be used to modify the functionality of food ingredients to achieve the desired properties of a specific food product. Furthermore, they found that the DBD treatment can change carbonyl content in fish protein ranging from 0.5–1.5 nmol/mg compared with the control samples to 0.5–2.5 nmol/mg of protein for the plasma treated samples. These features indicate that DBD treatment encourages protein oxidation, which leads to the development of crosslinking, which causes beef products’ juiciness, softness, and other qualitative attributes to decline (72). Besides, after a certain minute DBD treatment, the solubility of peanut protein isolate and the stability of emulsion increased (73–75) due to the solitary structure of peanut protein unfolding, which causes the content of -sheets and random coils to increase, while the content of -helixes and -turns decreases. The hydrophobic properties of whey protein also increased after being treated with low-pressure plasma (76).

### Cold plasma applied on lipids

Reactive oxygen species produced by the plasma process, including hydroxyl radicals, hydrogen peroxide, and superoxide anions, aid in the destruction of microorganisms. Unluckily, reactive species, especially free radicals, can start the oxidation of lipids by removing hydrogen ions from lipid molecules (77). It should be mentioned that despite the known negative effects on lipids, scientists are still actively investigating its use and developing innovative plasma sources for food applications (78).

**FIGURE 3:**
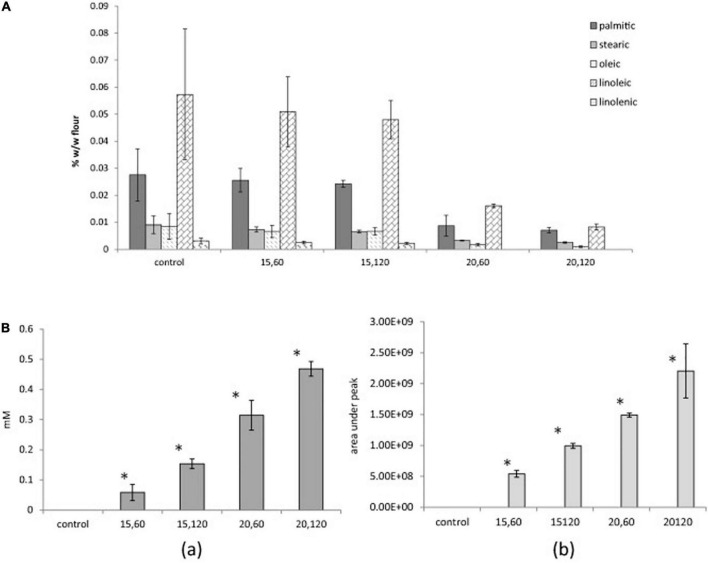
**(A)** Free fatty acids (FFAs) composition of control and cold plasma (CP)-treated wheat flour samples: 15, 60 (15 V, 60 s); 15, 120 (15 V, 120 s); 20, 60 (20 V, 60 s); and 20, 120 (20 V, 120 s). **(B)** Lipid oxidation markers (a) PV (hydroperoxide value) and (b) n-hexanal of control and CP-treated wheat flour samples: 15, 60 (15 V, 60 s); 15, 120 (15 V, 120 s); 20, 60 (20 V, 60 s); and 20, 120 (20 V, 120 s), *difference from control (*p* < 0.05) (80).

Sarangapani et al. (79) illustrated that with FTIR spectra and ^1^H NMR analysis, the hydroperoxides and aldehydes formed in butter oil and beef fat after 30-min DBD treatments. Bahrami et al. (80) considered that total free fatty acids and phospholipids were reduced by CP treatment; however, this effect was dose dependent. The rate of lipid oxidation was confirmed to be accelerated by the increase in oxidation markers (hydroperoxide value and head space n-hexanal) with treatment time and voltage by a specially designed CP system by a custom-made CP system, which confirmed the acceleration of lipid oxidation (Figure 3). The total number of aerobic bacteria or mould did not change as a result of the therapy. This was probably caused by the low levels of treatment and moisture (80).

Some researchers got the opposite results. Pérez-Andrés et al. (71) observed that after DBD treatment, neither the fatty acid composition nor the nutritional quality indices showed any significant changes (*p* > 0.05) in mackerel. Similar results were also obtained by Gavahian et al. (81); due to the modest penetration depth of plasma-generated reactive species, the characteristics of yolk (e.g., fatty acid composition, acid value, and thiobarbituric acid-reactive substances) were unaffected by plasma treatment.

### Cold plasma and functional components

Reactive oxygen species (ROS) and reactive nitrogen species (NOS) are created when the ambient air is used as a working gas. However, functional compounds (polyphenols, vitamins, etc.) may protect against oxidative stress by scavenging ROS, which may interact with bioactive substances, changing their quantity, and functional characteristics in food products and which might result in lipid peroxidation, protein oxidation, and DNA oxidation. The effects of plasma technology on the elements and characteristics of both solid and liquid foods have been the subject of numerous research. These applications primarily deal with the lowering of microbe and enzyme activity. Changes in the functional components of food following plasma therapy are therefore of particular relevance.

The antioxidant ability of sliced apples treated with DBD was investigated by Ramazzina et al. (82). The results indicated that DBD treatment caused only a slight reduction of antioxidant content and up to 10% antioxidant capacity. Moreover, in human cultured colonocytes, treated apple polyphenol extracts did not decrease cell viability and suppress the healthy physiological response of the cells to oxidative stress in terms of reactive oxygen species generation and phase II enzyme activation. Carotenoids, vitamin C, antioxidant activity, and angiotensin-converting enzyme (ACE) are all types of phenolic chemicals’ inhibitory activity in N2CP-treated guava-flavored whey beverage were investigated by Silveira et al. (62). Compared with thermal pasteurization, higher antioxidant activity, higher amounts of vitamin C, and volatile compounds were produced by CP treatment, but lower levels of carotenoids and a less favorable fatty acid profile were seen along with low N2 flow rates and short treating times (62). Similar results also can be found in CP-treated apple juice (83) and blueberry juice (44). These investigations showed that CP can be successfully used as a food processing technique without significantly degrading product quality.

## Properties of cold plasma treatment

Numerous reasons, such as the growing world population, which in turn raises the need for food, water, and energy resources, contribute to the increased demand for innovative sustainable technology in the agricultural and food sectors. The potential for CP technology to offer revolutionary and long-lasting technological interventions is illustrated by a large number of cases of inactivation of a variety of microorganisms and enzymes with proven efficacy for managing many dangers across these sectors. However, the risks of CP-treated food still need to be studied in detail, to prove that it cannot cause a negative impact on human health or the environment.

### Advantages of cold plasma technologies

These advantages of CP technologies include low-temperature operation, quick processing times, excellent energy economy, and strong antibacterial efficacy with little effect on environmental quality and food safety. Some investigations that looked at product consumption found no differences in the sensory acceptability of dried squid shreds treated with corona discharge for microbial decontamination despite losses in appearance, color, flavor, taste, and texture in water content and increased lipid peroxidation (84).

The production of ROS, such as ozone, may result in the bleaching of produced color and adverse effects on aesthetics. However, when exposed to plasma-activated water, fresh food such as tomatoes, carrots, and lettuce displayed minute but noticeable color changes (85).

Importantly, no appreciable effects of plasma treatment on the vitamin C content, pH, turbidity, or Brix of orange juice in a study of the nutritional aspects of the beverage were observed (86). For this evidence, CP technologies can be considered as a non-thermal and fast-food treatment, which shows little impact on food qualities including color, flavor, taste, texture, and nutrition.

### Cold plasma technologies on food safety and agriculture sustainability

Cold plasma technology has been explored for treatments of raw materials, intermediate, finished, or packaged products, and the processing equipment, facilities, and environment due to the abundance of its advantages.

Many of the ongoing safety, spoilage, and contamination challenges in the agricultural and food industries are caused by microbes. The question of how to perform microbial decontamination without causing quality deterioration is being still explored by scientists. Due to their potential role in non-thermal food processing, CP treatments of food products have grown in favor during the past 10 years. There is no denying that CP treatment is a flexible strategy with a track record of success in reducing a wide range of risks in the food and agricultural sustainability sectors. The CP-related research area can be classified as:

1. Microbes control and antimicrobial restrictions: A variety of microorganisms, including food pathogenic bacteria and fungi, spoilage microorganisms, and grains, seeds, and crops meant for sowing or storage have all been successfully inactivated using CP.

2. Mycotoxin degradation: Mycotoxins such as aflatoxin, which pose serious dangers to both human and animal health when they contaminate seeds, grains, or crops, have been successfully degraded using CP technology (87).

3. Insect control: The use of CP caused appreciable increases in larval and pupal mortality as well as a decline in adult emergence.

4. Biochemical reaction termination/enzyme inactivation: When L-alanine is directly exposed to argon plasma, the COOH group and CNH_2_ group are degraded. (88).

5. Food materials’ innovative applications: For instance, starch modification.

6. Lowering pesticide residues on a variety of substrates and for a variety of different organochlorine and organophosphorus pesticide chemicals (89).

### Hazard of cold plasma technologies

Many questions about CP need to be addressed due to the extent of the research that needs to be further developed. In general, the following questions need to be studied in-depth:

1. How the biological/chemical changes happened in food during or after CP treatment? As described, there have been several documented chemical modifications to dietary ingredients. Some of them would lose bioactivities after CP treatment. However, very limited evidence can be found that the by-products showed detrimental qualities to human health. For instance, sugars oxidize into organic acids; the modification of proteins, loss of protein structure, disruption of the a-helical structure into amino acid, and the peroxidation of lipids and unsaturated fatty acids were not clear.

2. What is the potential toxicity of CP-treated food? There were insufficient studies on the safety of plasma for use in culinary applications. The evaluations of the persistence of cytotoxic effectors of chlorine, ozone ROS/RNS, and nitrite (nitrate) that existed in CP-treated food should be carried out including their concentration and their oral toxicity.

3. Are there any effects of plasma on the human body directly? An interest in their application for cancer treatment has been spurred by cytotoxic action in mammalian cell models following CP treatments (90). Mutagenic effects of protein treated by cold atmospheric plasma have also been reported in some studies (91, 92). It is hard to perorate that CP would cause serious damage to the human body because of no evidence of the observed conviction. However, larger sample sizes are necessary to estimate the safety of CP treatments, and necessary shielding accessories are recommended during the design of CP devices.

4. What is the proper dosage of CP treatment? Certain power input is essential when concerning the performances of allergen control, microbial killing and enzyme inactivation, etc. Some concentrations of by-products, such as nitrite and ROS/RNS, depending on the plasma equipment and the treatment conditions, can approach the mM level. How to balance the performances and concentration of harmful compounds also needs to be determined.

### Aspects of cold plasma technologies on food processing

In recent years, several studies have successfully demonstrated food preservation using CP. CP technologies also can be applied in food-related areas, including modification of chemical structures and desensitization treatment. There is a need to thoroughly evaluate the advantages and disadvantages of stand-alone CP unit processes or their integration as a processing chain, as well as the advantages and acceptability of such processes for the economy, the environment, and consumers. To address the long-term and multigenerational impacts of plasma on seeds and plant growth during storage, further study is needed in the primary food production sector.

## Conclusion and recommendation of future research directions

A lot of studies have recently successfully shown how to store food using CP, including microbiological control and physiological and chemical quality characteristic modification. It also showed the potential of food by-product waste management and reducing immune reactivity by modifying the chemical structures. However, it still needs in-depth research on the facts as follows:

1. Can CP be used for controlling multispecies contaminants in complex food or environmental matrices?
2. Can food items become chemically contaminated as a result of CP deposition? Are there any by-products created by CP treatment that are harmful to human health?
3. What effects do organoleptic have on food composition? Does it have a good or any effect on taste?
4. How about the shelf-life profile of CP treated foods? Although CP can be used in controlling contaminants, the effect of food preservation still needs more investigation.

## Author contributions

HJ developed the idea of the study. HJ and QL designed the study and drafted the original manuscript. WS and XY searched the literature. SW and HJ critically revised and improved the manuscript. All authors contributed to the article and approved the submitted version.
